# Editorial: Unveiling novel aspects of SARS-CoV-2 to combat COVID-19

**DOI:** 10.3389/fgene.2024.1383640

**Published:** 2024-02-21

**Authors:** Nimisha Ghosh, Indrajit Saha, Dariusz Plewczynski

**Affiliations:** ^1^ Department of Computer Science and Information Technology, Institute of Technical Education and Research, Siksha ‘O’ Anusandhan (Deemed to be University), Bhubaneswar, India; ^2^ Institute for System Analysis and Computer Science “Antonio Ruberti”, National Research Council of Italy, Rome, Italy; ^3^ Department of Computer Science and Engineering, National Institute of Technical Teachers’ Training and Research, Kolkata, India; ^4^ Laboratory of Bioinformatics and Computational Genomics, Faculty of Mathematics and Information Science, Warsaw University of Technology, Warsaw, Poland; ^5^ Laboratory of Functional and Structural Genomics, Centre of New Technologies, University of Warsaw, Warsaw, Poland

**Keywords:** COVID-19, evolution, genome sequecing, protein synthesis, SARS-CoV-2

## 1 Introduction

Four years ago, the whole world was forced to come at a standstill due to SARS-CoV-2 virus, the causative agent of the COVID-19 pandemic. For more than 2 years, after incessant trials and tribulations by the scientific community and the common people alike, the spread of the virus and the deaths caused by the same are finally under control. This has been possible due to the efforts of the researchers which led to the development of several vaccines like Pfizer-BioNTech, Oxford-AstraZeneca, Novovax, Covaxin, Moderna (now called Spikevax), Comirnaty, Sputnik V, and Johnson & Johnson. However, in the initial days of the pandemic the knowledge about this deadly virus was very scarce. To mitigate this problem, whole genome analysis was carried out to better understand the virus. Thus, the knowledge that have been gained through rigorous research that is still ongoing may help to prevent such outbreaks in the future as well. Keeping all these factors in mind, in continuation of our earlier issue related to “SARS-CoV-2: From Genetic Variability to Vaccine Design”, we have further extended our service through the Research Topic “Unveiling novel aspects of SARS-CoV-2 to combat COVID-19” for the benefit of the scientific community. The articles covered in this Research Topic are discussed in subsequent sections.

## 2 Research topic organisation

The Research Topic is divided into three main sections: two papers delve into the evolution of SARS-CoV-2, two papers cover genome sequencing while two papers primarily focus on protein synthesis.

In the first part, the focus is on studying the evolution of SARS-CoV-2 and we believe that this section will appeal to researchers working in such area. In this regard, the first paper focuses on the adaptive evolution within Omicron variant of SARS-CoV-2 as collected from Bangladesh. The second paper on the other hand monitors the evolution of SARS-CoV-2 genome to identify insertions and deletions (indels) which remodel viral proteins’ surfaces resulting in adaptive selection.

The second part discusses genome sequencing and global gene expression. In this section, the first work performs whole genome sequencing using technique such as Oxford Nanopore Technology on genomic sequences from Kazakhstan to track viral variants in the country. The purpose of the second paper is to identify highly overexpressed genes and their functional implications due to COVID-19 infection in order to better understand the impact of coronavirus infection.

The third and final section encompasses the role of protein synthesis to fight against SARS-CoV-2 where the first work focuses on identifying potential human proteins that can be targeted by drugs to combat COVID-19 infection. The second paper works with cross-reactive T-cell recognition between common cold coronaviruses and SARS-CoV-2.

## 3 Evolution

Habib et al. have used 1711 SARS-CoV-2 Omicron genomic sequences from Bangladesh to study the corresponding adaptive evolution. Their study reveals 22 codon sites of the Spike gene displayed a sign of positive selection along with Membrane and ORF6 genes. Moreover, the receptor-binding motif within the receptor-binding domain (RBD) in Spike protein is the main point of adaptive evolution where some of these adaptive sites are also known to be associated with increased viral fitness. The results thus suggest that even though purifying selection may be the dominant evolutionary force, positive Darwinian selection should also be considered to have an important role in the evolution of Omicron variant in Bangladesh.

Alisoltani et al. have shown that with the advancement of the COVID-19 pandemic, numbers and ratio of genomes with in-frame insertions and deletions (indels) have increased significantly in the variants of concern indicating that indel distribution is correlated with spike mutations resulting in immune escape. The authors also show that indels are mostly present in hypervariable regions (HVRs) of individual SARS-CoV-2 proteins. Finally, they hypothesise that the increased indel frequency along with their non-random distribution and independent co-occurrence in several variants of concern is one of the ways by which the virus is responding to elevated immunity of global population.

## 4 Genome sequencing

Kairov et al. have used Oxford Nanopore Technologies (ONT) to sequence SARS-CoV-2 genomes in order to track viral variants circulating in Kazakhstan. In this regard, this is the first study that uses ONT approach for whole genome sequencing in Kazakhstan.

Jabeen et al. have tried to identify overexpressed genes and their functional implications as a result of COVID-19 infection. Moreover, they have also investigated probable infections, inflammation and immune system to better understand the impact of COVID-19. In this regard, they have identified genes such as NFKBIA, FN1, FAP, KANK4, COMP, FAM101B, COL1A2, ANKRD1, TAGLN, SPARC, ADAM19, OLFM4, CXCL10/11, OASL, FOS, APOBEC3A, IFI44L, IFI27, IFIT1, RSAD2, NDUFS1, SRSF6, HECTD1, CBX3, and DDX17 that may be affected by COVID-19. They have further explored potential herbal drug targets for the top-rated genes.

## 5 Protein synthesis

Tasneem et al. have identified therapeutic target proteins in human that can in turn act as drug targets against SARS-CoV-2. In this regard, they have employed structure-based similarity approach to predict human proteins similar to SARS-CoV-2 proteins which is then followed by identifying protein-protein interactions between the virus and its target human proteins. Furthermore, overlapping genes have also been identified between the protein-coding genes of the target and the mRNA expression data of COVID-19 infected patients. In this study, the authors have identified 19051 unique target human proteins that interact with SARS-CoV-2.

Cicaloni et al. have investigated the cross-reactive T-cell recognition between circulating common cold coronaviruses and SARS-CoV-2 encompassing variants such as Delta and Omega. They also used Siamese LSTM networks for aligning protein structures where the model is trained to calculate a BLAST-like similarity score between protein sequences.

